# Human Derived Exosome Injections (ASCE+ vs. CELLEXOSOME) Enhance Collagen Remodeling and Angiogenesis in Intact Skin: A Comparative Experimental Study

**DOI:** 10.1111/jocd.70787

**Published:** 2026-03-05

**Authors:** Noury Adel, Jack Kolenda, Francisco Llano, Jesper Thulesen, Francisco Hernandez Gomez Crespo, Yundun Shen, Youn Kyong Jo, Gabriela Yareli Zavala Resendiz, Diego Ivan Briseno Torres, Jesus Alejandro Lopez‐Lara, Ida Vega Thulesen

**Affiliations:** ^1^ Oral and Maxillofacial Surgery Specialist Private Practice Cairo Egypt; ^2^ Department of Otolaryngology Head and Neck Surgery University of Toronto Toronto Ontario Canada; ^3^ Private Practice Mexico; ^4^ Private Practice Copenhagen Denmark; ^5^ Eleve Clinic, Kirei University Mexico; ^6^ Orient Oculofacial Clinic Taipei Taiwan; ^7^ Orasyun Clinic South Korea; ^8^ Nuboskin Clinic, Kirei University Mexico; ^9^ Blux Aesthetic Center Mexico; ^10^ Eye Department Roskilde University Hospital Roskilde Denmark

**Keywords:** CD34, dermal regeneration, exosomes, extracelluar matrix, matriosome, secretomes, skin regeneration, skin rejuvenation

## Abstract

**Background:**

Exosomes derived from human cells have emerged as promising biological agents for enhancing skin quality through stimulation of collagen remodeling and angiogenesis. While their role in wound healing is well established, their effects on intact, non‐injured skin remain insufficiently investigated. Their biological activity depends on their molecular cargo, including growth factors, extracellular matrix‐modulating proteins, and angiogenic microRNAs.

**Objective:**

To evaluate the impact of intradermal injection of two human‐derived exosome formulations on dermal architecture and vascular density in intact skin.

**Methods:**

A total of 96 adult male Syrian golden hamsters were randomly assigned to four equal groups: untreated control, saline injection, Cell Exosome (0.1 mL), or ASCE+ Exosome (0.1 mL). Skin biopsies were collected at baseline, day 3, day 7, and day 14 post injection, with equal numbers of animals sacrificed per group and time point. Histological analyses (Hematoxylin & Eosin, Masson's Trichrome, Van Gieson) assessed dermal architecture and collagen organization, while CD34 immunohistochemistry quantified microvascular density. Quantitative image analysis was performed using ImageJ, with five high power fields evaluated per specimen. All assessments were performed in a blinded manner.

**Results:**

Untreated control and saline groups showed no significant histological or immunohistochemical changes across all time points, consistent with normal tissue architecture. Cell Exosome treatment produced moderate increases in collagen deposition and CD34 positive vessels. Quantitatively, ASCE+ increased collagen density and microvascular counts compared with Cell Exosome (*p* < 0.05), whereas control and saline groups showed no measurable changes.

**Conclusions:**

Human derived exosomes promote collagen remodeling and angiogenesis in intact skin, with ASCE+ Exosome exhibiting superior efficacy over Cell Exosome. These findings highlight the potential of exosome‐based therapies as minimally invasive strategies for skin rejuvenation.

**Abbreviations**
ANOVA: analysis of variance
ARRIVE: animal research: reporting of in vivo experiments
CD34: cluster of differentiation 34 (endothelial cell marker)
DAB: 3,3′‐diaminobenzidine
ECM: extracellular matrix
H&E: hematoxylin and eosin
HSD: honestly significant difference
IACUC: Institutional Animal Care and Use Committee
MSC: mesenchymal stem cell
MT: Masson's Trichrome

## 1 Introduction

Skin regeneration and rejuvenation represent central challenges in both dermatology and aesthetic medicine. While classical wound healing models focus on repairing tissue after injury, there is growing interest in enhancing the regenerative capacity of intact skin to maintain structural integrity, delay age related changes, and improve overall skin quality. Such strategies have important implications for preventive dermatology, cosmetic interventions, and minimally invasive regenerative therapies [1, 2, 3, 4, 5, 6, 7, 8, 9, 10].

Among emerging biologics, exosomes, nanosized extracellular vesicles secreted by diverse cell types, have attracted substantial attention as mediators of intercellular communication. By delivering proteins, lipids, and nucleic acids, exosomes regulate critical processes including fibroblast proliferation, extracellular matrix remodeling, angiogenesis, and immune modulation. In dermatology, exosomes derived from human cells have demonstrated the capacity to stimulate collagen synthesis, improve dermal architecture, and accelerate cutaneous repair. Their nanoscale dimensions also favor dermal penetration and cellular uptake, positioning them as highly promising candidates for skin revitalization beyond conventional wound healing models. Exosomes exert their biological effects by delivering proteins, cytokines, and extracellular matrix modulating microRNAs that regulate fibroblast activity, collagen homeostasis, and endothelial cell proliferation [11, 12, 13, 14, 15].

Human derived exosomes are typically isolated from mesenchymal stem cells, dermal fibroblasts, or other regenerative cell sources under controlled laboratory conditions. They contain a cargo of growth factors, cytokines, messenger RNAs, and microRNAs that regulate extracellular matrix turnover and neovascularization. Their mechanism of action is primarily paracrine: by transferring this bioactive content to recipient skin cells, they stimulate fibroblast proliferation, collagen synthesis, and endothelial sprouting, while also modulating inflammatory signaling. This multifaceted biological activity makes them uniquely suited for dermatologic applications, where both structural integrity and vascular support are essential for skin homeostasis and rejuvenation [12, 16, 17, 18].

Two exosome formulations have gained clinical interest: Cell Exosome Black Label Skin and ASCE+ Exosome (ExoCoBio, South Korea). Both are manufactured under standardized conditions to ensure stability and bioactivity, yet their relative efficacy in promoting dermal remodeling in intact skin has not been systematically compared. Notably, ASCE+ Exosome has been reported to contain enriched signaling molecules that may augment fibroblast activity and angiogenesis, though direct head‐to‐head evaluations in non‐injured skin remain limited [19, 20].

Importantly, different exosome formulations are not identical. Variations in the source cells, isolation methods, and stabilization techniques can significantly alter their molecular composition and regenerative potency. For example, ASCE+ Exosome is enriched in angiogenic and anti‐inflammatory factors, whereas other formulations such as Cell Exosome may contain higher proportions of extracellular matrix regulators. Synthetic or engineered exosome mimetics are also under development, designed to replicate exosomal signaling without the variability inherent to biological preparations. However, concerns remain regarding their bioactivity, stability, and potential immunogenicity compared with naturally secreted, human‐derived vesicles.

Unlike invasive resurfacing or filler based approaches, intradermal delivery of exosomes offers a minimally invasive intervention that may improve dermal physiology without inducing overt tissue injury. This distinction is critical, as intact skin models allow evaluation of regenerative enhancement under physiological conditions rather than reparative responses to wounding. Such an approach may better reflect clinical applications aimed at skin quality improvement, anti‐aging therapies, and preventive dermatologic care [21].

Despite the expanding literature on exosomes in wound repair, there is a lack of high quality evidence on their effects in normal, intact skin. Furthermore, it remains unclear whether all exosome formulations exert equivalent effects on collagen remodeling and angiogenesis, or whether specific preparations outperform others in promoting dermal health. This represents a crucial translational gap because skin rejuvenation procedures typically target healthy tissue; addressing this gap is essential for guiding translational applications and optimizing therapeutic protocols.

While human derived exosomes are generally well tolerated in preclinical and early clinical studies, potential safety considerations include immune reactions, off target signaling, or theoretical risks of promoting abnormal cell proliferation [22, 23, 24]. To date, no major adverse effects have been documented in dermatologic applications, but systematic studies in intact skin are lacking. Careful evaluation of histological and angiogenic responses in controlled models is therefore essential to confirm both the efficacy and safety profile of these biologic products before broad clinical translation.

The present study was therefore designed to evaluate, in a controlled animal model, the histological and immunohistochemical effects of intradermal injections of Cell Exosome and ASCE+ Exosome on intact skin. Using saline injection and untreated skin as controls, we assessed changes in dermal architecture, collagen deposition, and microvascular density across multiple time points. We hypothesized that exosome‐treated groups, particularly ASCE+, would demonstrate superior enhancements in collagen remodeling and angiogenesis compared with controls, thereby supporting their potential as novel interventions for skin rejuvenation.

## 2 Materials and Methods

### 2.1 Animals and Housing

Ninety six adult male Syrian golden hamsters ( *Mesocricetus auratus* ), weighing 100–120 g, were used in this study. Animals were housed under standardized laboratory conditions with controlled temperature (22°C ± 2°C), humidity (50% ± 10%), and a 12 h light/dark cycle, with ad libitum access to food and water. Animals were acclimatized for 7 days prior to experimentation. The experimental protocol was approved by an Institutional Animal Care and Use Committee (IACUC) and conducted in strict accordance with ARRIVE guidelines, ensuring humane handling and minimizing animal distress. Predefined humane endpoints included severe weight loss (> 20%), impaired mobility, or signs of distress, triggering early intervention. Syrian golden hamsters were selected due to the similarity of their dermal structure and collagen remodeling dynamics to human skin, providing a relevant model for translational dermatologic research.

### 2.2 Sample Size Determination and Power Analysis

Sample size was determined a priori using power analysis to detect a minimum 20% difference in collagen density and microvascular density between treatment groups, with a power of 0.85 and *α* = 0.05. Based on preliminary data, six animals per group per time point were required to achieve sufficient statistical power while minimizing animal use, in accordance with the 3Rs principle (Replacement, Reduction, Refinement). This ensured robust detection of treatment effects while maintaining ethical standards in animal research.

### 2.3 Randomization and Blinding

Animals were randomly assigned to one of four groups (*n* = 24 per group): untreated control, saline injection (0.1 mL), Cell Exosome injection (0.1 mL), or ASCE+ Exosome injection (0.1 mL). Randomization was performed using a computer generated sequence, and group allocation was concealed until the time of injection. All injections, sample collection, histological processing, imaging, and data analysis were conducted in a double blinded manner. Operators performing injections and assessments were independent of the study design team. Inter‐observer variability in histological and immunohistochemical assessments was quantified, and discrepancies were resolved by consensus.

### 2.4 Exosomes Products

Cell Exosome Black Label Skin (Abiomaterials Co., South Korea) and ASCE+ Exosome (ExcoBio, South Korea) were obtained as dual vial formulations, consisting of a lyophilized powder and a liquid vial for reconstitution immediately prior to use. Lot numbers, batch IDs, and storage conditions were recorded to ensure reproducibility. Products were handled according to manufacturer instructions, with injections performed promptly after mixing to preserve vesicle integrity. Both exosome formulations were administered at a dose of 0.1 mL intradermally. Sterile saline (0.1 mL) served as the vehicle control. Exosomes were stored and handled based on manufacturer instructions prior to administration to maintain stability.

### 2.5 Injection Procedure

General anesthesia was induced with intraperitoneal ketamine (80 mg/kg) and xylazine (10 mg/kg), ensuring adequate analgesia and immobility throughout the procedure. Animals were randomly allocated to one of four groups (*n* = 24 per group): untreated control, saline injection (0.1 mL), Cell Exosome injection (0.1 mL), or ASCE+ Exosome injection (0.1 mL). Each animal received a single intradermal injection in the mid dorsal region, delivered as a slow bolus over 3–5 s to prevent dermal rupture. Injection sites were marked with a sterile 2‐mm skin marker to guarantee consistent biopsy collection. Animals were monitored post injection for recovery, analgesic requirements, and overall well being. Biopsies were collected at baseline, day 3, day 7, and day 14 post injection, with six animals per group sacrificed at each time point. This design ensured balanced representation across groups and time points, enabling robust statistical analysis while minimizing biological variability. All procedures, including injections and subsequent assessments, were performed in a double blinded manner to reduce experimental bias.

### 2.6 Biopsy Collection and Histological Assessment

At designated time points, animals were humanely euthanized, and full thickness skin biopsies encompassing the epidermis and dermis were harvested from the injection sites. Samples were fixed in 10% neutral buffered formalin, paraffin embedded, and sectioned at 5 μm thickness. Histological evaluation was conducted using Hematoxylin and Eosin (H&E) staining to assess tissue architecture, cellularity, and inflammatory infiltration, following standard protocols (Sigma Aldrich, USA). Collagen deposition and organization were analyzed using Masson's Trichrome (HT15 kit, Sigma Aldrich) and Van Gieson staining (ab150666, Abcam), providing complementary confirmation of dermal remodeling.

Angiogenic activity was assessed via immunohistochemical staining for CD34. Sections were incubated with rabbit monoclonal anti CD34 antibody (Abcam ab81289, 1:200). Antigen retrieval was performed in citrate buffer (pH 6.0) at 95°C for 20 min, followed by blocking with 5% bovine serum albumin for 30 min. Staining was visualized using the DAB chromogen detection system (Dako EnVision) and counterstained with hematoxylin. Microvascular density was quantified by counting CD34 positive endothelial cells in five randomly selected high power fields per section using ImageJ software (NIH, USA). All slides were coded by an independent technician, and evaluations were conducted by two board certified dermatopathologists in a double blinded manner. Interobserver discrepancies were resolved by consensus to ensure reliable data assessment.

### 2.7 Statistical Analysis

Data are expressed as mean ± standard deviation. Normality and homogeneity of variance were assessed using the Shapiro Wilk and Levene's tests, respectively. Intergroup comparisons at each time point were performed using one way analysis of variance (ANOVA), followed by Tukey's Honestly Significant Difference (HSD) post hoc test for multiple comparisons. Statistical significance was set at a two sided *p* value < 0.05. All analyses were performed using IBM SPSS Statistics version 28 (IBM Corp., Armonk, NY, USA). Missing data, if any, were handled using listwise deletion.

## 3 Results

All animals tolerated the procedures without adverse effects. Daily observations confirmed normal behavior, grooming, and food/water intake. No local or systemic reactions (ulceration, necrosis, inflammation, or allergy) were observed at injection sites, indicating that both human derived exosome formulations and saline injections were biocompatible and safe for intradermal administration.

### 3.1 Histological Evaluation (Hematoxylin & Eosin)

H&E stained sections of untreated control and saline injected skin consistently demonstrated normal epidermal and dermal architecture across all time points. Epidermal thickness, dermal cellularity, and the presence of fibroblasts remained stable, and there was no evidence of inflammatory cell infiltration, confirming that saline injections did not alter baseline skin histology.

Cell Exosome treated skin exhibited early dermal cellular activation by day 3, characterized by scattered fibroblast proliferation and a slight increase in dermal cellularity. By day 7, fibroblast density had moderately increased, with some early extracellular matrix deposition noted. At day 14, the dermal–epidermal interface remained intact, fibroblast proliferation was maintained, and collagen accumulation was evident, though dermal organization was only moderately improved compared with control.

**FIGURE 1:**
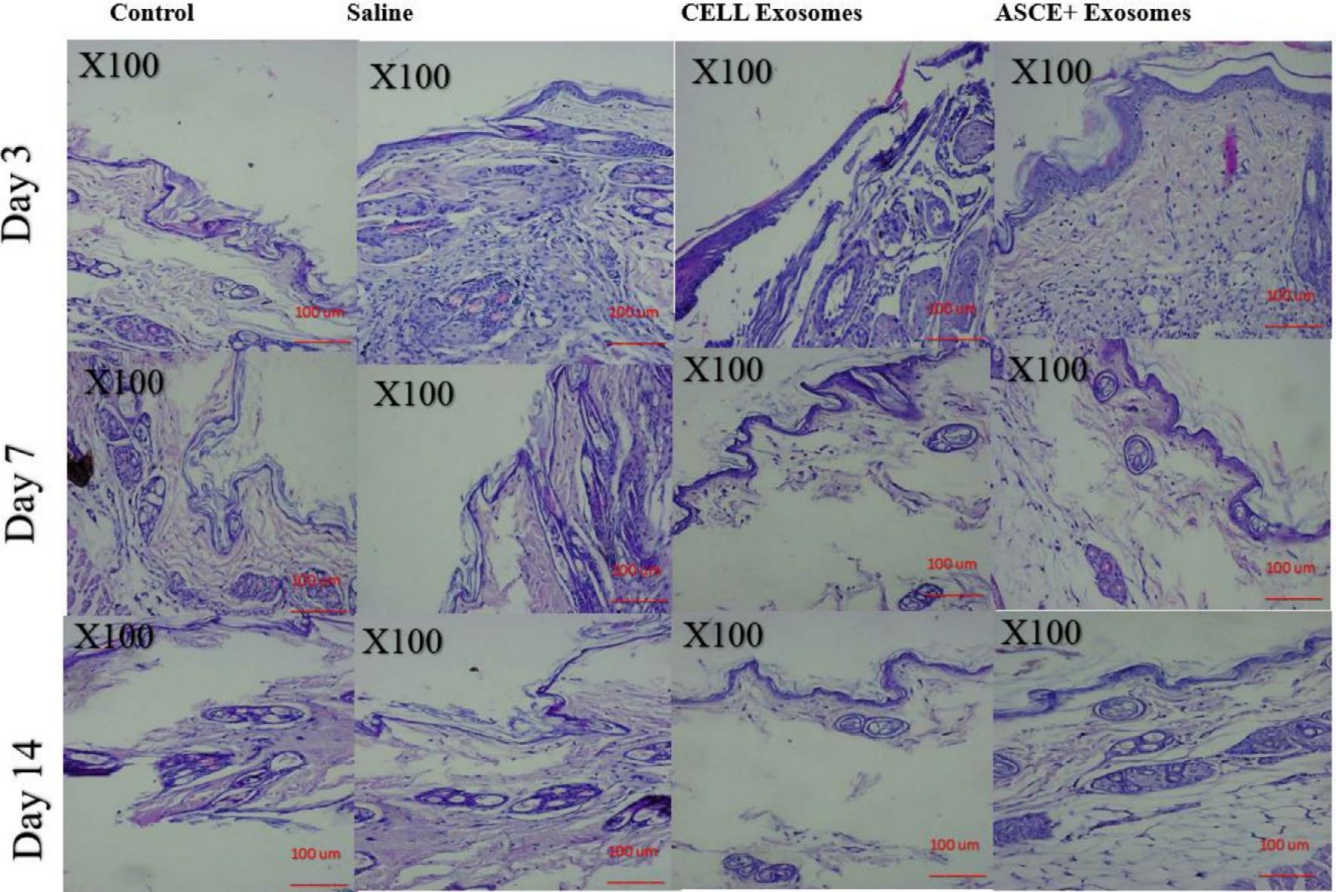
Hematoxylin & Eosin stained skin sections at days 3, 7, and 14 across treatment groups. Representative H&E images show that control and saline groups maintained normal epidermal and dermal architecture at all time points. Cell Exosome–treated skin exhibited mild fibroblast activation and increased dermal cellularity beginning at day 3 and progressing through day 14. ASCE+ Exosome–treated skin demonstrated the most pronounced response, with early fibroblast activation at day 3, marked dermal cellularity and matrix deposition at day 7, and well‐organized dermal structure by day 14.

ASCE+ Exosome treated skin displayed more pronounced histological changes. At day 3, early fibroblast activation and an increase in dermal cellularity were apparent. By day 7, fibroblasts were densely packed, and extracellular matrix deposition was markedly increased. At day 14, ASCE+ Exosome treated skin demonstrated highly organized dermal architecture, with dense fibroblast populations, clear dermal–epidermal cohesion, and minimal inflammatory infiltrates, indicating accelerated tissue remodeling relative to Cell Exosome and control groups (Figure 1).

### 3.2 Collagen Deposition and Organization (Masson's Trichrome and Van Gieson Staining)

Masson's Trichrome and Van Gieson staining revealed minimal collagen changes in control and saline groups at all time points. Collagen fibers were thin, loosely arranged, and maintained the baseline pattern typical of intact skin.

Cell Exosome treated skin showed moderate increases in collagen deposition by day 7, with fibers appearing thicker and partially aligned. By day 14, collagen bundles were more densely packed and slightly better organized, though they lacked the uniform alignment observed in ASCE+ Exosome treated tissue.

**FIGURE 2:**
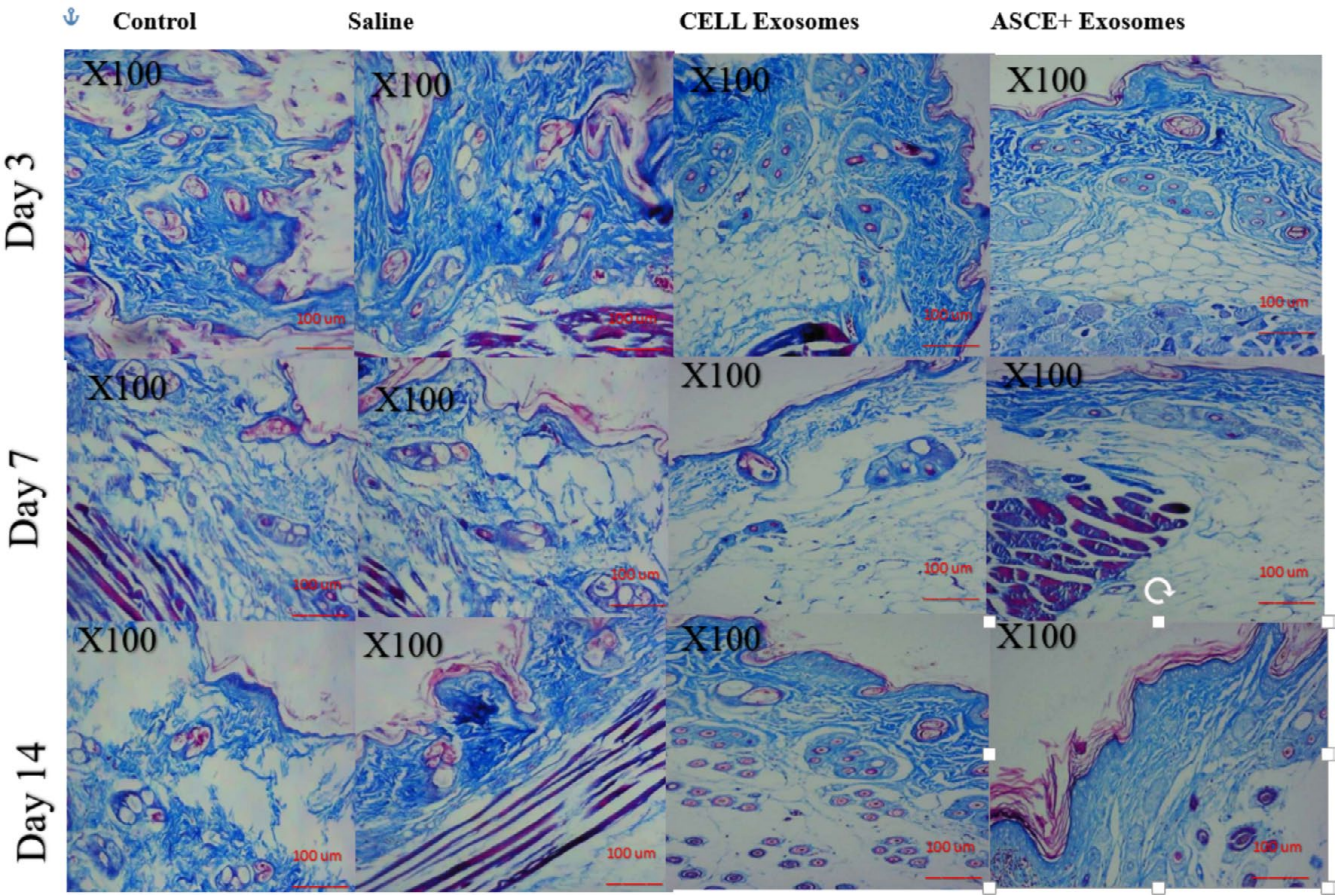
Masson's Trichrome staining of skin sections at days 3, 7, and 14 across treatment groups. MT staining shows minimal collagen changes in control and saline groups. Cell Exosome treated skin demonstrates progressive increases in collagen thickness and partial fiber alignment. ASCE+ Exosome treatment produces the strongest response, with early collagen deposition at day 3, dense and aligned collagen bundles by day 7, and markedly organized collagen architecture by day 14.

ASCE+ Exosome treatment resulted in robust collagen remodeling. At day 3, early collagen deposition was evident in the superficial dermis. By day 7, collagen bundles were thick, dense, and well aligned, forming a more organized network throughout the dermis. At day 14, extensive, tightly packed collagen bundles were present, confirming superior structural remodeling. Van Gieson staining corroborated these findings, highlighting increased collagen density and improved fiber alignment compared with all other groups (Figure 2).

### 3.3 Angiogenesis (CD34 Immunohistochemistry)

CD34 immunostaining revealed minimal microvascular structures in control and saline groups at all time points, consistent with baseline dermal vascularity.

Cell Exosome treated skin demonstrated a moderate increase in CD34 positive endothelial cells, particularly by day 7, suggesting early angiogenic stimulation. This moderate elevation persisted at day 14 but did not reach the levels observed in ASCE+ Exosome treated skin.

**FIGURE 3:**
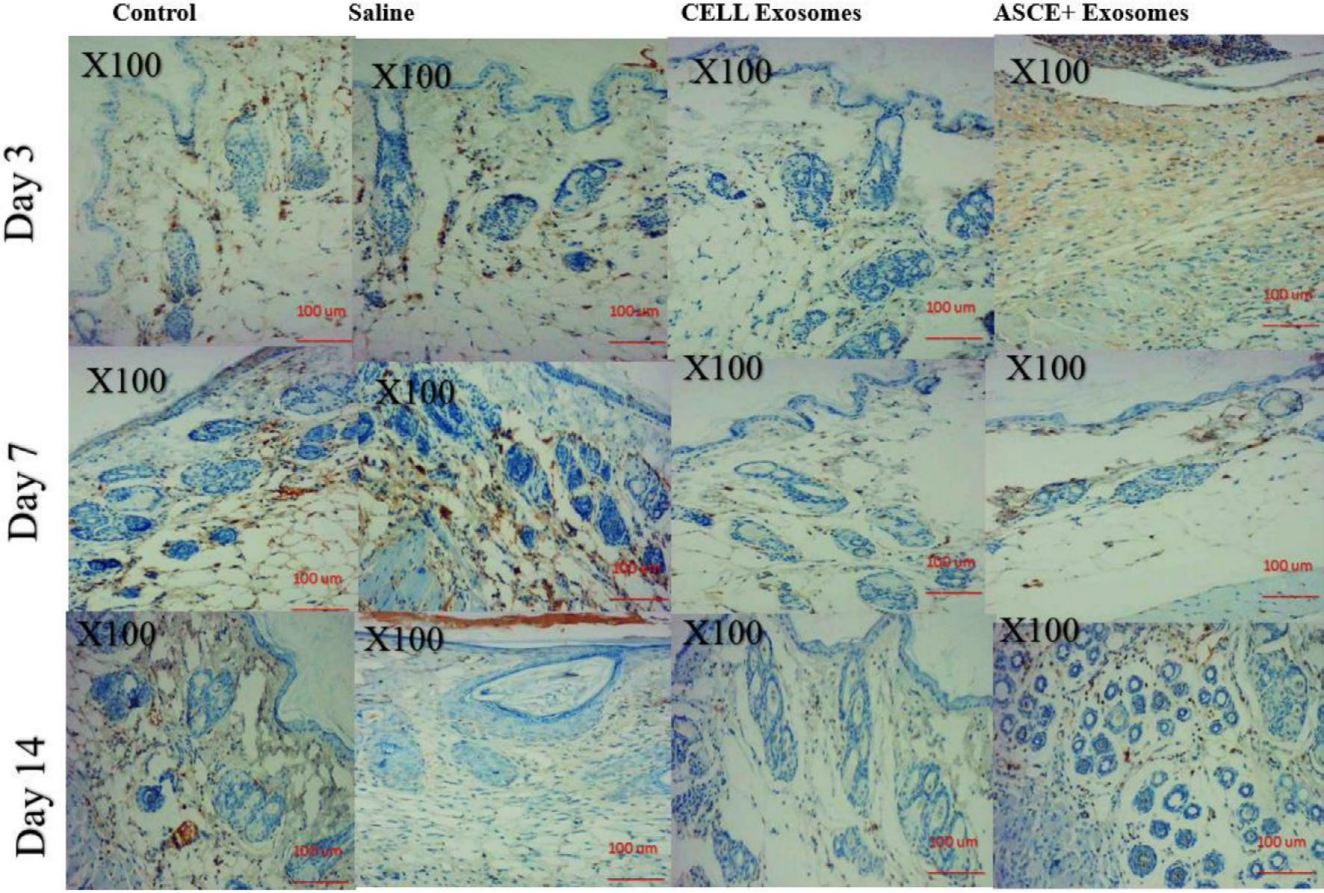
CD34 immunohistochemical staining of dermal microvasculature at days 3, 7, and 14 across treatment groups. CD34 staining reveals no vascular changes in control or saline groups. Cell Exosome–treated skin shows a moderate increase in CD34 positive microvessels beginning at day 3. ASCE+ Exosome treatment induces a robust angiogenic response, with dense CD34 positive vascular networks at days 7 and 14, representing the highest microvascular density among all groups.

ASCE+ Exosome markedly enhanced angiogenesis. By day 3, small clusters of CD34 positive vessels were visible in the dermis. Day 7 sections showed proliferation of endothelial cells forming dense microvascular networks, and by day 14, ASCE+ Exosome treated skin exhibited the highest microvascular density among all groups, with numerous mature CD34 positive vessels distributed throughout the dermis. These findings indicate that ASCE+ Exosome effectively promotes neovascularization in intact skin, surpassing the angiogenic effect of Cell Exosome (Figure 3).

**FIGURE 4:**
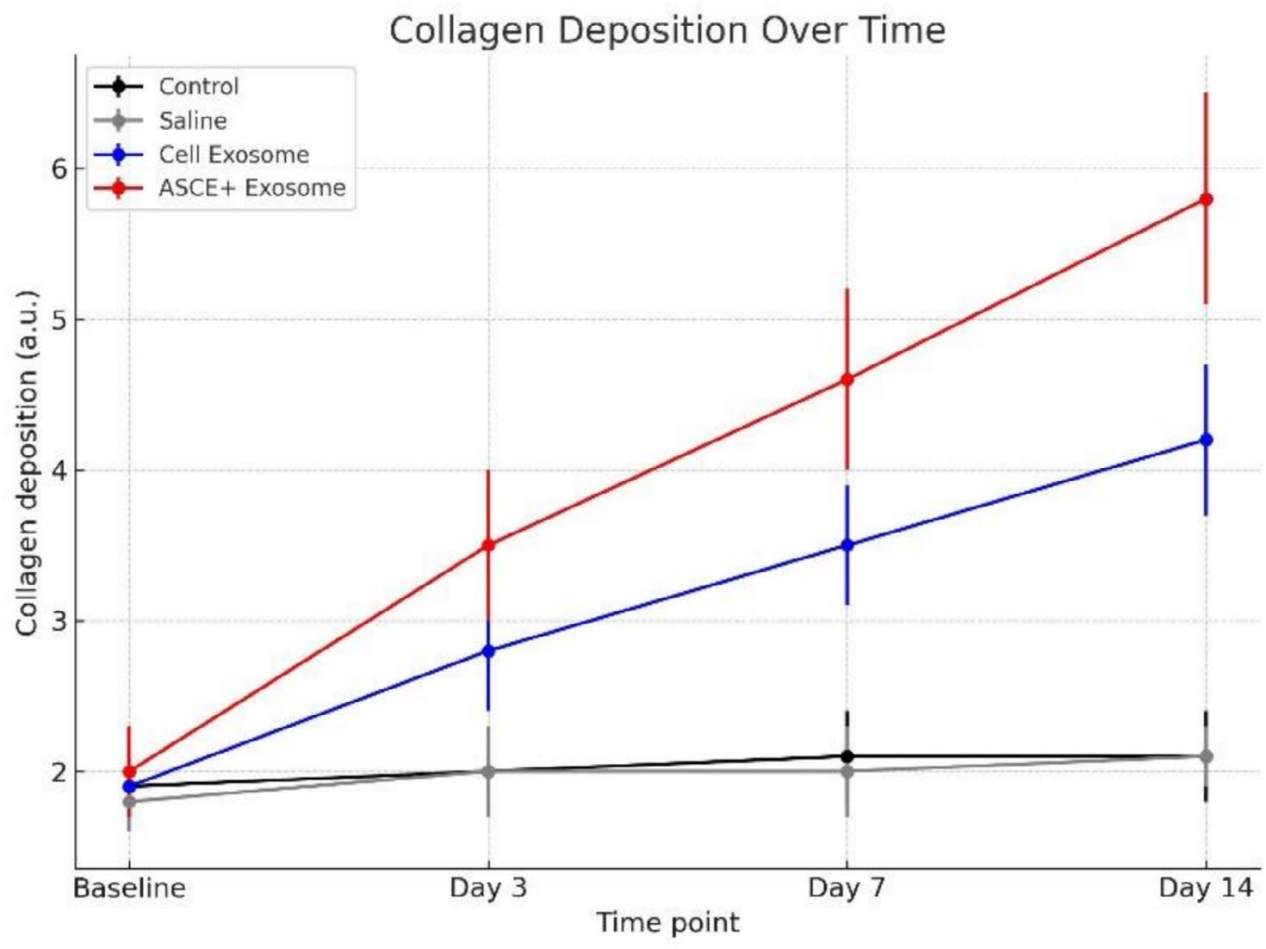
Collagen deposition in intact hamster skin following intradermal injections of saline, Cell Exosome, or ASCE+ Exosome at baseline and days 3, 7, and 14. Data are expressed as mean ± standard deviation. Collagen fiber density remained unchanged in control and saline groups. Both exosome treated groups exhibited progressive increases over time. ASCE+ Exosome produced the most significant and sustained enhancement compared with Cell Exosome and controls. Intergroup differences at each time point were analyzed using one‐way ANOVA with Tukey's post hoc test.

**FIGURE 5:**
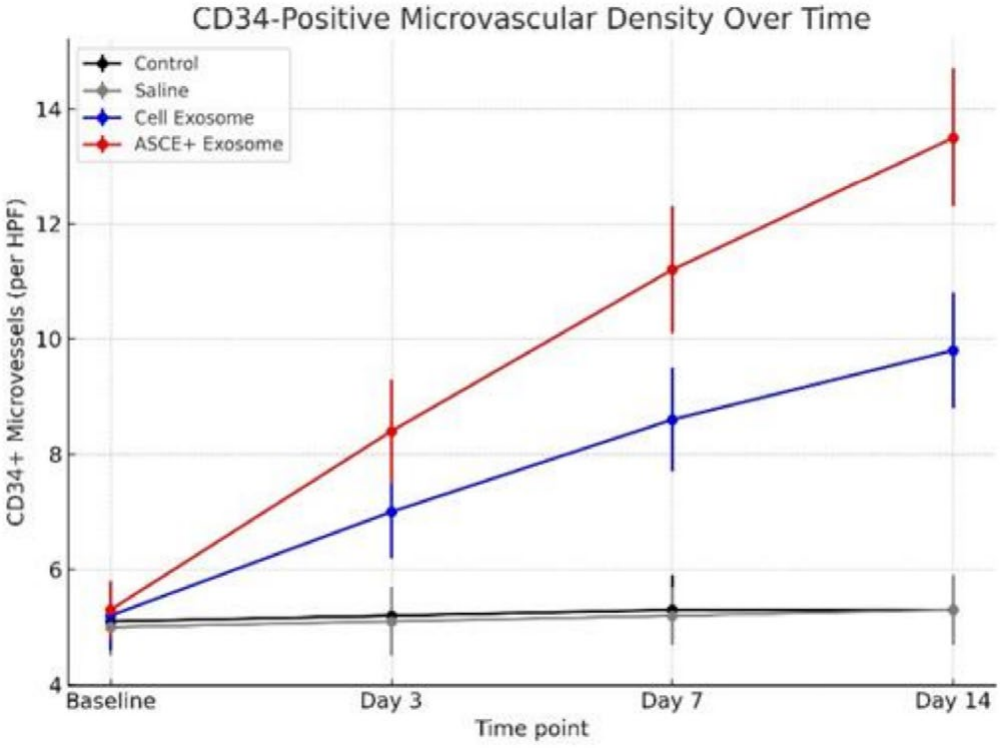
CD34 positive microvascular density in intact hamster skin after intradermal injection of saline, Cell Exosome, or ASCE+ Exosome at baseline and days 3, 7, and 14. Data are presented as mean ± standard deviation (vessels per high power field). Minimal changes were observed in control and saline groups. Both exosome treated groups showed significant temporal increases in microvascular density. ASCE+ Exosome produced the highest angiogenic response. Intergroup differences at each time point were analyzed using one way ANOVA with Tukey's post hoc test.

Overall, the comparative analysis across groups revealed that exosome therapy promoted structural and vascular remodeling in intact skin in a treatment dependent manner. The ASCE+ Exosome group consistently produced the highest fibroblast activation, collagen fiber density, and CD34 positive microvascular counts, while Cell Exosome induced moderate but measurable improvements. In contrast, saline and untreated controls showed no significant histological or immunohistochemical changes over the entire study period. These findings suggest that human derived exosomes, particularly ASCE+, exert a potent regenerative effect in non injured skin (Tables 1 and 2; Figures 4 and 5).

**TABLE 1:** Quantitative assessment of collagen deposition in intact hamster skin following intradermal injections of saline, Cell Exosome, or ASCE+ Exosome at baseline and days 3, 7, and 14.

Time point	Control	Saline	Cell Exosome	ASCE+ Exosome	*p*‐value (ANOVA)	Significance (Tukey HSD)
Baseline	1.9 ± 0.3	1.8 ± 0.2	1.9 ± 0.2	2.0 ± 0.3	0.92	NS (no difference)
Day 3	2.0 ± 0.3	2.0 ± 0.3	2.8 ± 0.4	3.5 ± 0.5	0.012	ASCE+ > Cell Exosome > Control/Saline
Day 7	2.1 ± 0.3	2.0 ± 0.3	3.5 ± 0.4	4.6 ± 0.6	< 0.001	ASCE+ > Cell Exosome > Control/Saline
Day 14	2.1 ± 0.3	2.1 ± 0.2	4.2 ± 0.5	5.8 ± 0.7	< 0.001	ASCE+ ≫ Cell Exosome > Control/Saline

*Note:* Data are presented as mean ± standard deviation (arbitrary units). Intergroup differences at each time point were assessed using one‐way ANOVA with Tukey's post hoc test. Significant increases in collagen fiber density were observed over time in exosome‐treated groups (*p* < 0.05), with ASCE+ Exosome producing the greatest enhancement compared with Cell Exosome and controls.

**TABLE 2:** Quantitative evaluation of CD34 positive microvascular density in intact hamster skin following intradermal injection of saline, Cell Exosome, or ASCE+ Exosome at baseline and days 3, 7, and 14.

Time point	Control	Saline	Cell Exosome	ASCE+ Exosome	*p*‐value (ANOVA)	Significance (Tukey HSD)
Baseline	5.1 ± 0.6	5.0 ± 0.5	5.2 ± 0.6	5.3 ± 0.5	0.88	NS (no difference)
Day 3	5.2 ± 0.5	5.1 ± 0.6	7.0 ± 0.8	8.4 ± 0.9	0.015	ASCE+ > Cell Exosome > Control/Saline
Day 7	5.3 ± 0.6	5.2 ± 0.5	8.6 ± 0.9	11.2 ± 1.1	< 0.001	ASCE+ > Cell Exosome > Control/Saline
Day 14	5.3 ± 0.5	5.3 ± 0.6	9.8 ± 1.0	13.5 ± 1.2	< 0.001	ASCE+ ≫ Cell Exosome > Control/Saline

*Note:* Data are presented as mean ± standard deviation (vessels per high power field). Intergroup differences at each time point were analyzed using one way ANOVA with Tukey's post hoc test.

## 4 Discussion

Tissue regeneration and dermal remodeling remain central objectives in both aesthetic dermatology and regenerative medicine. Strategies that enhance fibroblast activation, collagen synthesis, and neovascularization are of particular interest for improving skin quality and delaying age‐related degeneration [25, 26]. In this study, we investigated the effects of human‐derived exosomes on intact hamster skin, comparing Cell Exosome and ASCE+ Exosome formulations against saline and untreated controls. Our findings demonstrate that ASCE+ Exosome produced the most pronounced improvements in collagen deposition and microvascular density, while Cell Exosome also yielded measurable, though less robust, effects.

The safety profile of all tested interventions was favorable. No systemic or local adverse events were observed, including inflammation, necrosis, or infection. This absence of toxicity highlights the biocompatibility of human derived exosomes, an important consideration for their potential clinical application in minimally invasive dermatologic procedures.

Histological analysis provided further insight into the regenerative processes induced by exosome therapy. Hematoxylin and Eosin sections revealed increased fibroblast activity and improved dermal organization in both exosome‐treated groups compared with controls, with the ASCE+ Exosome group showing the greatest enhancement. By day 14, these changes translated into thicker dermal layers, reduced inflammatory cell presence, and more cohesive dermal–epidermal junctions, suggesting a progressive remodeling effect.

Specialized collagen stains (Masson's Trichrome and Van Gieson) confirmed that exosome therapy enhanced extracellular matrix deposition and organization. The ASCE+ Exosome group exhibited abundant, densely packed, and well aligned collagen fibers, indicating accelerated fibroblast‐driven synthesis and maturation of connective tissue. Cell Exosome also improved collagen deposition compared with controls, but the effect was less pronounced, reflecting potential differences in exosomal cargo or potency.

Angiogenic activity, assessed by CD34 immunohistochemistry, paralleled these findings. Both exosome formulations increased microvascular density, with ASCE+ Exosome demonstrating the strongest proangiogenic response. Enhanced vascularity likely contributes to improved nutrient and oxygen delivery, as well as paracrine support for fibroblast activity, thereby reinforcing collagen deposition and dermal regeneration. These results are consistent with prior reports showing that exosomes stimulate endothelial cell proliferation and migration through delivery of proangiogenic proteins and microRNAs.

The observed superiority of ASCE+ Exosome compared with Cell Exosome may be attributed to compositional differences in vesicle content, manufacturing processes, or donor cell sources. Exosomes are known to carry diverse bioactive molecules, including growth factors, cytokines, and nucleic acids, whose relative abundance influences their regenerative efficacy. Further comparative proteomic and transcriptomic analyses are needed to clarify the mechanisms underlying these differences.

This study has several methodological strengths, including standardized animal allocation, blinded histological assessment, and evaluation at multiple time points to capture the dynamics of dermal response. Nevertheless, some limitations must be acknowledged. The follow up period was relatively short, preventing conclusions about the long term persistence of collagen and vascular changes. Functional parameters such as skin elasticity or biomechanical strength were not assessed, and molecular analyses were not performed to directly validate mechanistic pathways. Additionally, although hamsters provide a reproducible model, caution should be exercised when extrapolating findings to human skin.

Taken together, our findings suggest that human‐derived exosomes, particularly the ASCE+ formulation, can safely and effectively enhance dermal regeneration in intact skin through stimulation of fibroblast activity, collagen deposition, and angiogenesis. These results support the potential translational value of exosome‐based therapies in cosmetic dermatology and regenerative interventions. Future studies with extended follow‐up, functional endpoints, and molecular characterization are warranted to optimize clinical protocols and better define the therapeutic role of exosomes in skin rejuvenation.

## 5 Conclusion

Intradermal administration of human‐derived exosomes enhances collagen remodeling and angiogenesis in intact skin, with ASCE+ Exosome demonstrating superior regenerative efficacy compared with Cell Exosome. These results support the use of exosome‐based therapies as safe, minimally invasive strategies for skin rejuvenation.

## Author Contributions

Noury Adel was responsible for conceptualization, conceived the study concept and design, performed the experimental procedures, data analysis, manuscript writing and drafting; the rest of the authors gave a hand to manuscript drafting and revision. All authors approve the final submitted version and agree to be accountable for all aspects of the work.

## Funding

The authors have nothing to report.

## Ethics Statement

This study was conducted in accordance with the ARRIVE Guidelines for animal use. Ethical approval was obtained from an Institutional Animal Care and Use Committee.

## Conflicts of Interest

The authors declare no conflicts of interest.

## Data Availability

The datasets generated and analyzed during the current study are available from the corresponding author upon reasonable request and following appropriate ethical and regulatory procedures.
